# Efficacy of Laser Therapy in Comparison With Other Methods for the Treatment of Onychomycosis: A Systematic Review and Meta-Analysis

**DOI:** 10.7759/cureus.59720

**Published:** 2024-05-06

**Authors:** Christopher R Meretsky, Brooke L Friday, Anthony T Schiuma

**Affiliations:** 1 Surgery, St. George's University School of Medicine, Great River, USA; 2 Medical School, St. George's University School of Medicine, Great River, USA; 3 Orthopedic Surgery, Holy Cross Hospital, Fort Lauderdale, USA

**Keywords:** meta-analysis, systematic review, efficacy, terbinafine, laser therapy, onychomycosis

## Abstract

Onychomycosis, a fungal infection of the nails, presents a significant challenge in clinical management due to its chronic nature and resistance to conventional therapies. This study aims to evaluate the efficacy of laser therapy in treating onychomycosis compared to traditional methods such as terbinafine. A systematic review and meta-analysis were conducted to analyze existing literature on the subject. The Preferred Reporting Items for Systematic Reviews and Meta-Analyses (PRISMA) diagram illustrates the selection process of studies. Findings suggest that laser therapy demonstrates promising results in the treatment of onychomycosis, with comparable efficacy to terbinafine and fewer adverse effects. Further large-scale randomized controlled trials are warranted to validate these findings and establish laser therapy as a standard treatment option for onychomycosis.

## Introduction and background

Onychomycosis is a chronic fungal nail infection that causes tissue damage to the nail matrix or plate bed. It is estimated to account for more than half of all nail diseases [1]. This fungal infection-linked disease has become a significant public health issue due to poor response to treatment, frequent relapses, and high prevalence that led to significant social, financial, and clinical impacts. It affects larger populations globally, resulting in functional impairment, pain, and cosmetic disfigurement [2]. Although it is not a life-threatening clinical event, it is difficult to treat. Principally, the infection of nails is caused by yeasts, non-dermatophyte molds, and dermatophytes such as *Candida albicans*, *Trichophyton mentagrophytes*, and *Trichophyton rubrum* [3]. The clinical diagnosis of onychomycosis is performed by identifying the specific site of infection and type of infection. The various types of infection include proximal subungual onychomycosis, total dystrophic onychomycosis, superficial white onychomycosis, and distal or lateral subungual onychomycosis. Among those types, lateral and distal subungual onychomycosis is the most diagnosed [4,5]. 

In the last two decades, the prevalence of onychomycosis has increased and attributed to different factors such as the frequent use of occlusive modern footwear, increased urbanizations, longer life expectancies, and an increase in the number of immunocompromised patients [6,7]. Additionally, onychomycosis can cause foot issues, attributed to other conditions, such as diabetes and, in severe cases, can even require the removal of the infected toenails or foot parts [8]. Common risk factors behind the prognosis of onychomycosis are the history of fungal infection on any other body part, wearing occlusive footwear, the occurrence of nail psoriasis or nail injuries, and frequent participation in activities such as swimming and running [9]. 

Onychomycosis is a type of contagious fungal infection, primarily transmitted via contact of skin to skin or skin to the area having infected dead nail cells or skin cells and fomites having fungal propagules [10]. The proliferation of fungal infection secondarily may occur within the same person, infecting other nails, toes, and web spaces, leading to infection of the whole feet [11]. The untreated nail infection may lead to other skin manifestations such as atopic dermatitis, erythema nodosum, dermatitis, and other fungal infections of other body parts [12]. Various treatments of onychomycosis have been rapidly emerging, involving mechanical, oral, topical, and chemical treatment strategies. The type of treatment is suggested based on the severity of nail damage, type of fungal infection-causing agent, possible adverse effects and drug interactions, and failure or success ratio of previous cases [13]. The severity or extent of fungal infection can be detected by the degree of nail discoloration, nail involvement, onycholysis (extent of separation of the nail from the nail bed), pain, and nail plate thickening [14].

Most of these treatments are provided by systemic agents taken orally or topical agents applied directly to nails. Topical drugs such as amorolfine and ciclopirox are applied to nail plates for the management of minor infections [15]. Furthermore, newer topical agents like tavaborole 5% and efinaconazole 10% have shown better clinical outcomes as compared to placebo, for the treatment of onychomycosis [16]. These agents cause fewer side effects and drug interaction issues in the human body. However, topical drug treatments have not proved successful due to the inability of penetration in nail plates and longer treatment periods. However, topical antifungal therapies may be more successful when paired with chemical dissolution or surgical removal of the nail plate [14,15]. On the other hand, oral antifungals such as fluconazole, itraconazole, and terbinafine are reported to be highly effective for treating onychomycosis due to their capability of penetration in nail plates and nail beds [17]. Oral antifungal drugs might cause adverse reactions due to a high risk of hepatic and renal damage as well as potential drug interactions. Other side effects of oral agents are gastrointestinal symptoms, rashes, headaches, and nausea [17]. 

Terbinafine is an emerging and mostly recommended oral treatment strategy against onychomycosis. It is a topically and orally active antifungal agent, usually targeting the ergosterol of fungal cell membranes to inhibit the biosynthesis of sterol in fungi. The excellent fungicidal activity of terbinafine has been reported against yeasts, non-dermatophytes, and dermatophytes in vitro [18]. Terbinafine quickly gets absorbed and disseminated throughout tissues of the human body, such as the weakly perfused nail matrix, after oral treatment. Within one week of initiating therapy, nail terbinafine concentrations are found, and these effects continue for at least 30 weeks (about seven months) following treatment's end. About 10.5% of receivers of terbinafine suffered adverse effects, with stomach problems being the most frequent [19,20]. 

Considering these adverse events, laser therapy has been suggested as another option for onychomycosis treatment. Dermatological lasers have been utilized to treat a wide range of medical and cosmetic skin conditions. Researchers argue that these lasers provide a practical treatment with few adverse effects. Since the treatment is given in a clinical setting, patient adherence is not necessary [21]. Additionally, patients experiencing adverse events of systemic antifungals that can result in drug-drug interactions may benefit from laser therapy. Additionally, individuals with diabetes, older individuals with drug intolerance, and patients with liver and kidney problems may find that laser treatment is a more effective treatment option for their condition [22]. Laser systems have emerged as a modern treatment option against onychomycosis, rather than topical and oral drugs. The laser radiation restricts the growth of fungus by selective photothermolysis [23]. The laser treatment was initiated by Apfelberg in 1984 for the treatment of onychomycosis and was approved by the US Food and Drug Administration. Recent used laser treatments include long-pulsed 1064-nm neodymium-doped yttrium aluminum garnet (Nd:YAG) lasers, as well as short-pulsed 1064-nm Nd:CO2 lasers, and lasers with wavelengths of 870 nm, 930 nm, and 1320 nm. The adverse events after laser treatment are fewer such as bleeding and pain in the area around the infected nail [23-25]. 

Westerberg and Voyack [26] reported a 61% success ratio of laser treatment against onychomycosis at 16 weeks. About 91% of clinical efficacy and 30% success rate of laser treatment have been reported after 180 days. Previous studies [22-25] found that laser treatments are clinically effective for the treatment of onychomycosis by using a meta-analysis approach. However, studies evaluating the efficacy and safety of laser treatment in comparison to other traditional treatments such as terbinafine are limited. Therefore, the recent study aimed to evaluate the clinical outcomes and adverse events of laser treatments as compared to other treatment strategies against onychomycosis by using a systematic review and meta-analysis approach. It was predicted that the results of a recent study would guide future clinical implications of the most effective treatment against onychomycosis.

## Review

Materials and methods

The Preferred Reporting Items for Systematic Reviews and Meta-Analyses (PRISMA) guidelines were followed for conducting a recent systematic review and meta-analysis [27]. Since all the data for the recent study was collected from trials that were published, no additional ethical approval was required.

Search Strategy

**Figure 1:**
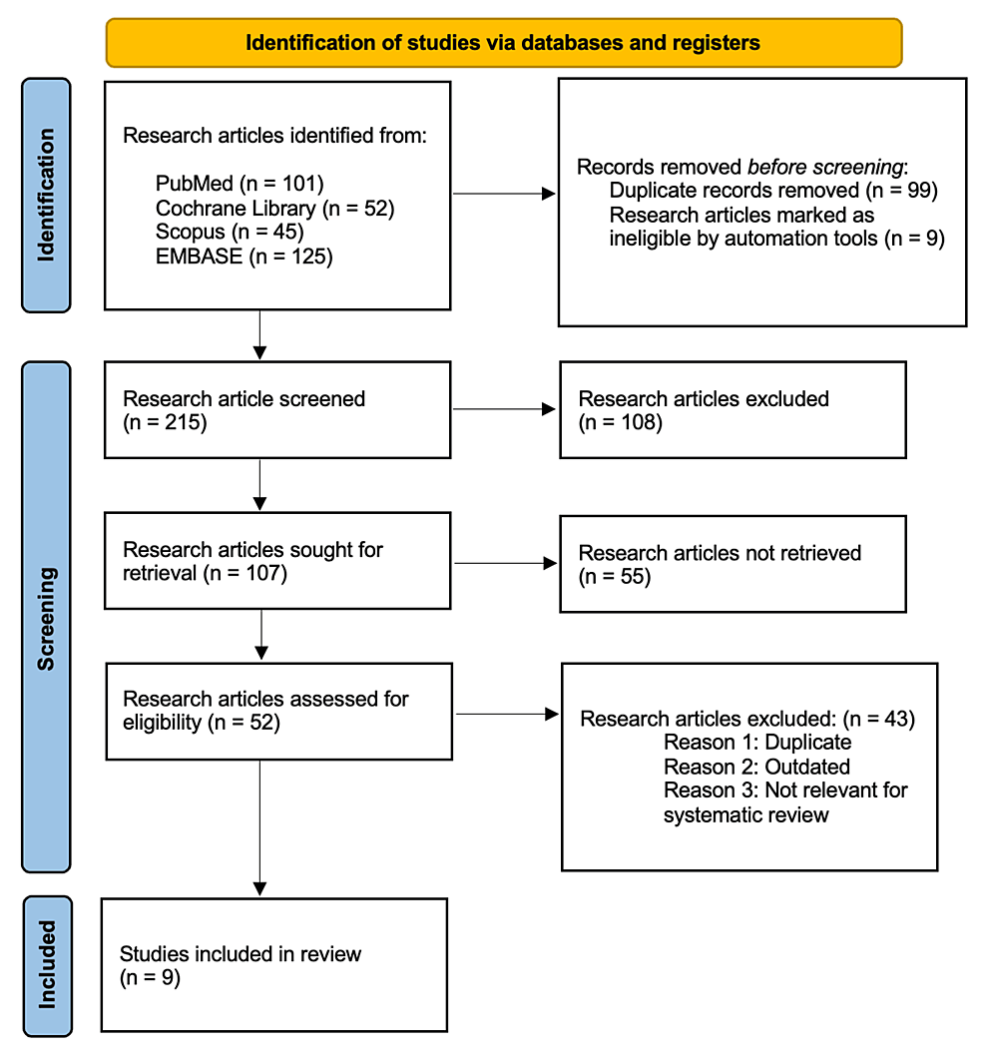
PRISMA flowchart: literature search and study selection n: number; PRISMA: Preferred Reporting Items for Systematic Reviews and Meta-Analyses

In a recent study, the research articles related to the study aim "Efficacy of laser therapy in the treatment of onychomycosis compared to other methods" were extracted from different databases, according to PRISMA guidelines [28]. Four electronic databases such as PubMed, Cochrane Library, Scopus, and Embase were used for data extraction. The Medical Subject Headings (MeSH) terms were used to reach authentic data, and these were ("Onychomycosis" OR "nail fungal infection" OR "fungal infection of nail" OR "infected or fragile nails") AND ("Laser therapy" OR "Laser treatment" OR "Photo radiation treatment") AND ("conventional treatments" OR "Terbinafine" OR "Oral treatment" OR Topical treatment") AND ("effectiveness" OR "Safety" OR "efficacy" OR "Adverse events"). A combination of these MeSH terms was used in the literature search. The timeline of research was set from January 2004 to January 2024. Articles published in peer-reviewed journals and relevant medical guidelines were included in the review. The study selection process is illustrated in Figure 1.

Criteria for Selection

The recent study was conducted by following the PICO (population, intervention, comparison, and outcomes) model, as shown in Table 1.

**Table 1:** Inclusion and exclusion criteria for the screening of studies based on the PICO model PICO: population, intervention, comparison, and outcomes

Study characteristics	Inclusion criteria	Exclusion criteria
Population	Adult population diagnosed with onychomycosis disease	Adult population with other nail infections or participants with non-onychomycosis disease
Intervention	Laser treatment against onychomycosis	Other treatment strategies such as topical agents against onychomycosis
Comparison	Laser treatment versus terbinafine treatment against onychomycosis	Laser treatment versus placebo or other treatment against onychomycosis
Outcomes	Mycological cure rates, clinical improvement, and adverse effects	No primary or secondary outcomes of interest

Inclusion criteria: The selection criteria assisted in the screening of research articles. In a recent study, only those articles that met the following criteria were included: (1) studies involving the adult population diagnosed with onychomycosis, (2) studies involving laser treatment versus terbinafine against onychomycosis, (3) studies discussing the outcomes related to the incidence of mycological cure rates, clinical improvement, and adverse effects, (4) studies based on randomized controlled trials (RCTs), pilot studies, and cohort studies and (5) studies that are published in English and where the full text is available.

Exclusion criteria: Only those studies that have the following features were excluded: (1) studies that discussed populations with other types of fungal infection rather than onychomycosis, (2) studies involving other treatment strategies rather than laser treatment such as topical and oral agents, (3) studies that discussed outcomes rather than incidence of mycological cure rates, clinical improvement, and adverse effects, (4) already published systematic reviews, meta-analyses, scoping reviews, literature reviews, conferences, and letters, and (5) studies that were published in other languages (such as Chinese, Spanish, Arabic, and German) and duplicated publications or non-full-text papers.

Timeline of the Study

The extraction and screening of research articles with pooled analysis was conducted from March 30, 2024, to April 15, 2024. 

Data Extraction 

After the selection of research articles from databases, the screening process was conducted in two phases. Firstly, the titles and abstracts of all research articles were studied after selection from chosen electronic databases. The list of research articles was compiled after the first phase for possible inclusion. Secondly, the complete texts of articles from the first round of screening were examined to reach authentic data [28]. For each eligible paper, we extracted the information related to authors, year of study, country, study population, sample size, type of laser treatment, study design, and primary outcomes such as mycological cure rates, clinical improvement, and adverse effects from selected articles after the selection and screening of research articles. 

*Risk of Bias Assessment* 

The Cochrane risk of bias tool was applied to examine the risk of bias of included RCTs [29,30]. The bias was examined on the basis of five domains: (a) allocation concealment, (b) selection bias or random sequence generation, (c) performance bias or blinding of participants and personnel, (d) detection bias or blinding of outcome assessment, and (e) selective bias or selective reporting and other bias. Each domain's score was categorized into high risk, unclear, and low risk. For comparative cohort studies, the MINORS scale [31] was applied to assess the quality of included articles. There are 11 items in the MINORS checklist to examine the quality of methodological aspects including a well-defined objective, inclusion of population who completed follow-up, prospective data collection, findings suitable to study objective, unbiased findings of study findings, loss to follow-up less than 5% prospective estimation of required sample size, appropriate follow-up period, baseline equivalence of population groups, a proper control group, contemporary groups, and adequate statistical analysis. The first eight items were linked to the methodological assessment of non-RCTs, and each item of the checklists was scored from 0 to 2.

*Statistical Analysis* 

In a recent systematic review and meta-analysis, the Review Manager (RevMan) software version 5.4.0 was used to conduct a pooled analysis of outcome data extracted from included studies [30]. The results of odds ratio (OR) with a 95% confidence interval (CI) were considered statistically significant with a p-value of <0.05. Furthermore, the heterogeneity was measured by using I2 statistics and the Q test. In case of no significant difference, the random effects model was applied for the calculation of OR.

Results

*Included Studies* 

The selection and screening of research papers according to the research aim "Efficacy of laser therapy in the treatment of onychomycosis compared to other methods" was conducted by following the PRISMA guidelines in the recent meta-analysis and systematic review. About 323 research articles were extracted from four electronic databases PubMed (n=101), Cochrane Library (n=52), Scopus (n=45), and Embase (n=125) after applying the abovementioned search strategy. By following the PRISMA guidelines [3], only 215 papers were screened, and 108 articles were excluded before screening. Among those, 107 articles were assessed for eligibility criteria, and the final number of research articles after applying exclusion criteria was 9.

Risk of Bias Assessment

**Figure 2:**
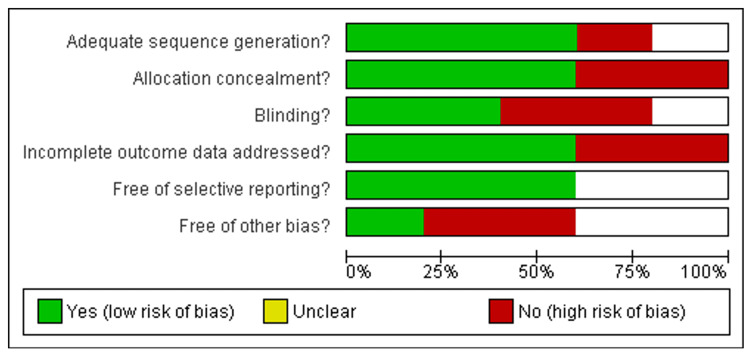
Risk bias graph of included studies

**Figure 3:**
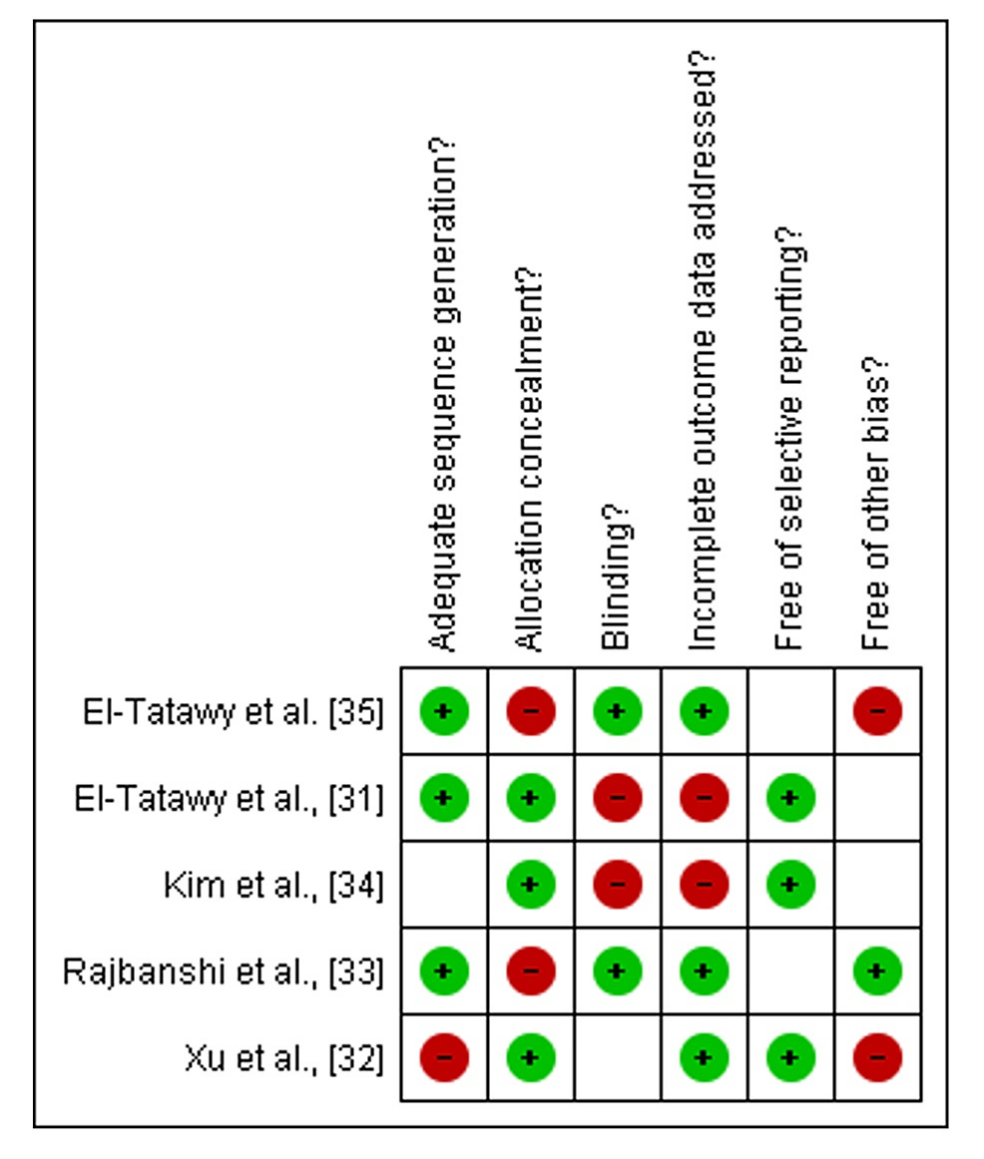
Graph of risk bias summary of included studies References: [31-35]

Among the nine included studies, five were RCTs [31-37], assessed by the Cochrane Library tool. About three out of five were low- to moderate-risk studies [32-34], and two studies were high risk [31,35] as shown in Figure 2 and Figure 3.

*Quality Assessment of Included Studies* 

In Table 2, the methodological quality of four included studies (non-RCTs and comparative studies) of recent studies was assessed by the MINORS checklist [31]. Only one study had moderate risk [38], while the other three studies were low risk [36,37,39].

**Table 2:** The quality assessment of included comparative cohort studies

Standards of checklist	Kartik and Kohli, 2022 [36]	Shetty et al., 2023 [37]	Koren et al., 2018 [38]	Lu et al., 2016 [39]
Clear and well-defined objective	2	2	2	2
Inclusion of population who completed follow-up	2	1	0	2
Prospective data collection	0	2	2	2
Conclusion suitable to study objective	1	2	2	0
Unbiased findings of study findings	2	2	1	1
Loss to follow-up less than 5%	2	0	0	2
Prospective estimation of required sample size	2	1	2	2
Accurate follow-up period	2	2	2	2
Equal baseline characteristics of population groups	2	2	1	1
Proper control group	1	2	2	2
Proper intervention group	2	2	2	2
Accurate statistical analysis	2	2	1	2
Total	20/24	20/24	17/24	20/24

Study Characteristics

About 533 individuals with onychomycosis were analyzed in a recent systematic review and meta-analysis to conclude. These research trials belong to five different countries: three from China [32,33,39], two from Egypt [31,35], one from Korea [34], two from India [36,37], and one from Israel [38]. The different types of drugs against laser therapy such as amorolfine, topical tioconazole, and terbinafine were used in all included studies and shown in Table 3.

**Table 3:** Characteristics of included studies

Author, year	Country	Study population	Sample size	Study follow-up	Study design	Type of treatment	Mycological cure rates	Clinical improvement	Adverse event
El-Tatawy et al., 2015 [31]	Egypt	40 patients with onychomycosis	20 in the laser treatment group and 20 in the terbinafine group	6 months	Randomized controlled trial	Laser treatment: 4 sessions. Terbinafine: twice daily	Laser treatment: 16 out of 20. Terbinafine: 10 out of 20	13 out of 20; 7 out of 20	Laser treatment pain: 10 out of 20. Terbinafine: 0 out of 20
Xu et al., 2014 [32]	China	53 individuals with onychomycosis	23 in the laser treatment group and 20 in the terbinafine group	24 weeks	Randomized controlled trial	Long-pulsed Nd:YAG laser and oral terbinafine	17 out of 23 in laser treatment; 16 out of 20 in terbinafine	15 out of 23 in laser treatment; 14 out of 20 in terbinafine	0 out of 23 in laser treatment; 2 out of 20 in terbinafine
Rajbanshi et al., 2020 [33]	China	160 individuals with onychomycosis	80 in the laser treatment group and 80 in the terbinafine group	6 months	Randomized controlled trial	Laser treatment, terbinafine	32 out of 80; 19 out of 80	18 out of 80; 3 out of 80	Nil
Kim et al., 2016 [34]	Korea	56 individuals with onychomycosis	36 in the laser treatment group and 18 in the terbinafine group	24 weeks or 6 months	Randomized controlled trial	1064 nm (Nd:YAG) laser treatment and topical treatment by terbinafine	26 out of 36; 3 out of 18	28 out of 36; 4 out of 18	Nil
El-Tatawy et al. 2019 [35]	Egypt	30 individuals with onychomycosis	20 in the laser treatment group and 10 in the topical tioconazole group	6 months	Randomized controlled trial	CO2 laser versus topical tioconazole	18 out of 20; 3 out of 10	19 out of 20; 4 out of 10	Nil
Kartik and Kohli, 2022 [36]	India	50 individuals	25 in the laser treatment group and 25 in the topical antifungal treatment group	6 months	Prospective study	Fractional CO2 versus terbinafine	20 out of 25; 11 out of 25		
Shetty et al., 2023 [37]	India	50 patients with fingernail onychomycosis	25 in the laser treatment group and 25 in the oral itraconazole pulse therapy group	4 months	Cohort study	Fractional CO2 laser therapy versus oral itraconazole pulse therapy	18 out of 25; 10 out of 25	14 out of 25; 6 out of 25	
Koren et al., 2018 [38]	Israel	60 individuals with onychomycosis	30 in the laser treatment group and 30 in the amorolfine group	6 months	Open-label comparative study	Fractional ablative CO2 laser versus amorolfine	25 out of 30; 13 out of 30		3 out of 30; 1 out of 30
Lu et al., 2016 [39]	China	34 individuals with onychomycosis	25 in the laser treatment group and 11 in the 5% amorolfine group	12 weeks	Pilot study	Laser treatment versus 5% amorolfine	10 out of 25; 8 out of 11	10 out of 25; 6 out of 11	

*Primary Outcomes* 

**Figure 4:**
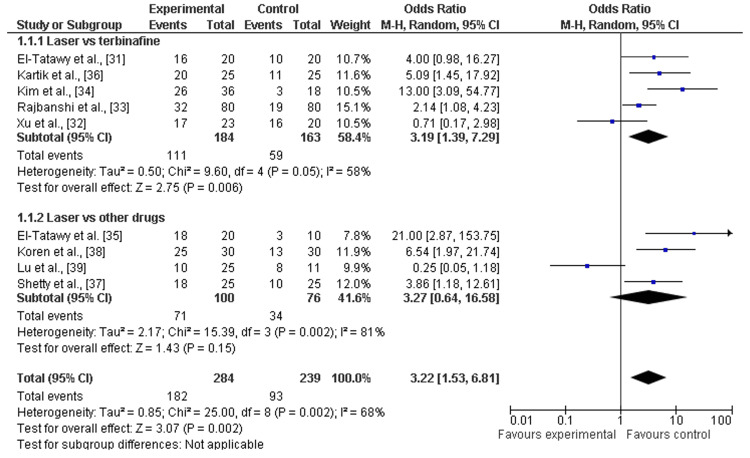
Forest plot of mycological cure rates among laser therapy and terbinafine or other drugs References: [31-39]

**Figure 5:**
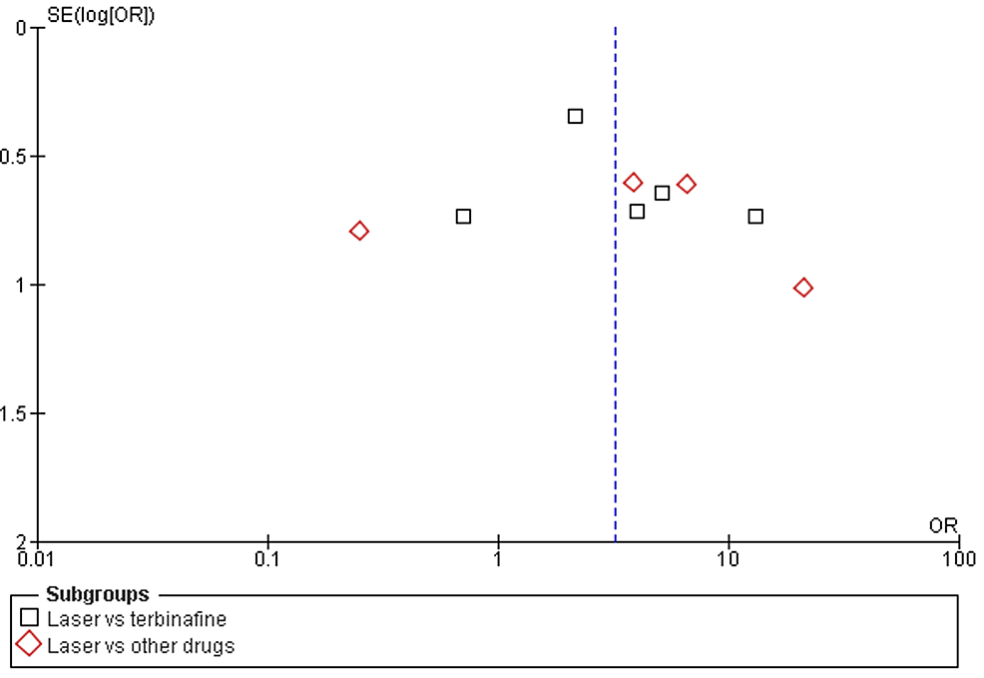
Funnel plot of mycological cure rates among laser therapy and terbinafine or other drugs

Mycological cure rates: Among the eight included studies, almost all studies discussed the mycological cure rates after a minimum of 12 weeks (about three months) and a maximum of 24 weeks (about six months) follow-up of laser therapy and terbinafine or other drugs (amorolfine and itraconazole pulse therapy) [31,32,38]. There was a significant increase in mycological cure rates after laser therapy as compared to terbinafine (OR=3.19; 95% CI: 1.39-7.29; p>0.05) or another drug (OR=3.13; 95% CI: 1.39-37.34; p>0.05), and heterogeneity was found (df=8; I2=68%), as shown in Figure 4 and Figure 5.

**Figure 6:**
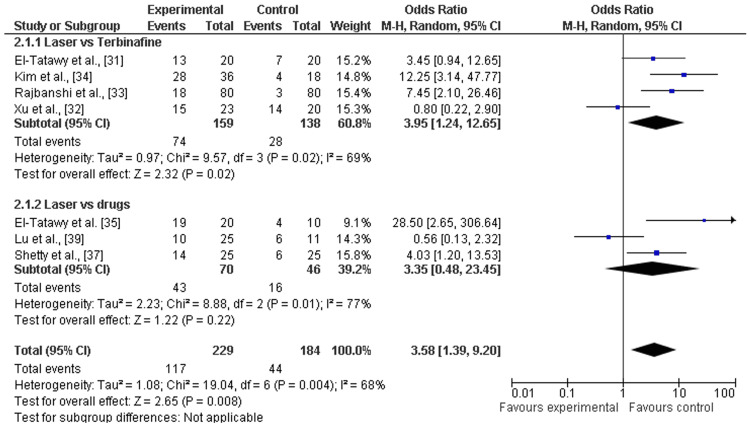
Forest plot of clinical cure rates among laser therapy and terbinafine or other drugs References: [31-35,37,39]

**Figure 7:**
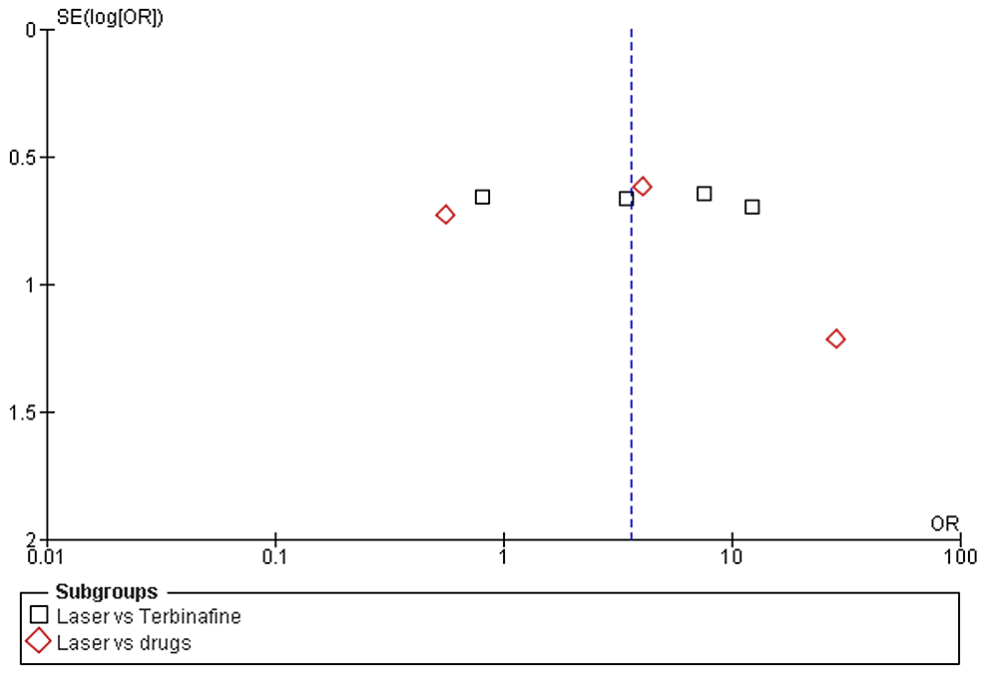
Funnel plot of clinical cure rates among laser therapy and terbinafine or other drugs

Clinical improvement: Among the eight included studies, almost seven studies discussed the clinical cure rates after a minimum of 12 weeks (about three months) and a maximum of 24 weeks (about six months) follow-up of laser therapy and terbinafine or other drugs (amorolfine and itraconazole pulse therapy) [31-35,37,39]. There was a significant increase in clinical cure rates after laser therapy as compared to terbinafine (OR=3.95; 95% CI: 1.24-12.65; p=0.02) or other drugs (OR=3.35; 95% CI: 0.48-23.34; p>0.05), and heterogeneity was found (df=6; I2=68%), as shown in Figure 6 and Figure 7.

**Figure 8:**
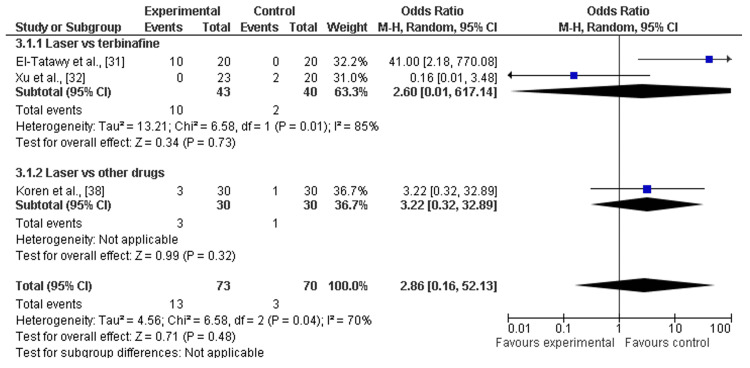
Forest plot of adverse events among laser therapy and terbinafine or other drugs References: [31,32,38]

**Figure 9:**
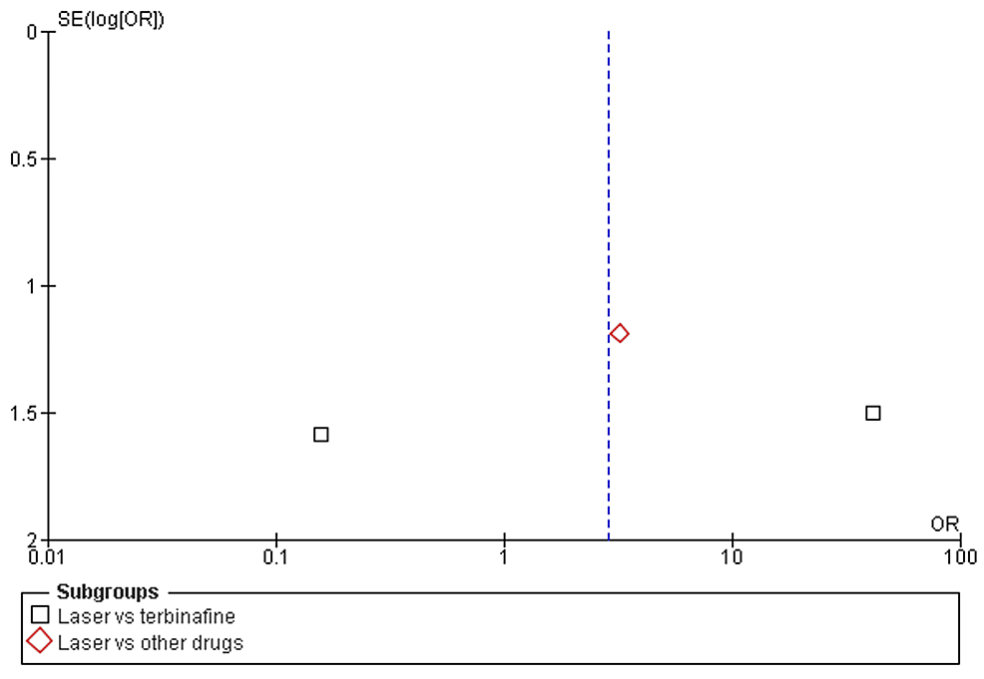
Funnel plot of adverse events among laser therapy and terbinafine or other drugs

Adverse events: Among the eight included studies, three studies discussed the adverse events after a minimum of 12 weeks (about three months) and a maximum of 24 weeks (about six months) follow-ups of laser therapy and terbinafine or other drugs (amorolfine and itraconazole pulse therapy) [31,32,38]. There was a significant increase in adverse events after laser therapy as compared to terbinafine (OR=2.60; 95% CI: 0.01-617.14; p>0.01) or other drugs (OR=3.22; 95% CI: 0.32-32.89; p>0.34), and heterogeneity was found (df=2; I2=70%), as shown in Figure 8 and Figure 9.

Discussion

In this study, the recent systematic review and meta-analysis were performed to assess the clinical effectiveness and safety (adverse events) of laser therapy in comparison to other methods (e.g., terbinafine, amorolfine, and itraconazole) for the treatment of onychomycosis. To fulfill research aims, we analyzed the data of 533 individuals and 1538 diseased nails with onychomycosis from five RCTs [31-35] and four comparative cohort studies [36-39]. The risk of selective reporting of included RCTs was assessed as low [31-35] and included cohort comparative studies showed moderate risks [36-39]. Most of the studies included were scored as being of moderate quality or low risk. In general, the pooled analysis revealed that laser treatment proved effective for the treatment of onychomycosis as compared to terbinafine and other drugs (such as amorolfine and itraconazole). Additionally, mycological cure rates were higher among participants receiving laser therapy as compared to participants receiving terbinafine (OR=3.19; 95% CI: 1.39-7.29; p>0.05) and other drugs (OR=3.13; 95% CI: 1.39-37.34; p>0.05) during a minimum of three months and a maximum of six months follow up [31-39]. Among participants receiving laser therapy as compared to terbinafine or the drugs, the clinical cure rates were higher, explained as laser therapy versus terbinafine (OR=3.95; 95% CI: 1.24-12.65; p=0.02) and other drugs (OR=3.35; 95% CI: 0.48-23.34; p>0.05). However, other topical or oral antifungal drugs result in fewer or no adverse events as compared to laser therapies that have higher risks or hazards as most adverse events are linked with the use of it for the treatment of diseased nails by onychomycosis. The higher rates of adverse events after laser therapy as compared to terbinafine (OR=2.60; 95% CI: 0.01-617.14; p>0.01) and other drugs (OR=3.22; 95% CI: 0.32-32.89; p>0.34) were reported as shown in Figure 8 and Figure 9. Thus, the overall effectiveness of laser treatment was comparatively higher than that of traditional topical antifungal treatments, but it resulted in higher rates of adverse events, such as pain, bleeding, mild redness, and feeling of burning [40]. Furthermore, the pooled analysis suggested laser treatment as a more suitable strategy for the treatment of onychomycosis as compared to terbinafine and other drugs, but less safe for different population subgroups such as children. 

Among subgroups, the OR values of laser therapy versus terbinafine were much stronger as compared to laser therapy versus topical drugs such as amorolfine and itraconazole for both mycological cure rates [31-39] and clinical cure rates [31-35,37,39]. However, only one study [38] demonstrated and reported the adverse events in subgroup laser versus other drugs. Hence, it is difficult to report the rates of adverse events among laser therapy versus other drugs. Terbinafine is an effective and safe therapy for onychomycosis in high-risk populations. It was successful and well-tolerable in diabetic people [41]. It is extremely effective in managing dermatophyte infections and was nearly the initial active treatment for onychomycosis. TBF-HCl blocks the enzyme squalene epoxidase in fungal ergosterol production, increasing intracellular squalene and premature death of cells [42].

Several studies reported the clinical outcomes of fractional carbon dioxide laser, diode laser, and long-pulsed neodymium-doped yttrium aluminum garnet (Nd:YAG) laser for the treatment of onychomycosis [14]. FDA-approved lasers are extensively recommended for the treatment of diseased nails by onychomycosis. It is hypothesized that lasers can be fungicides by photothermolysis, with rapid temperature increase resulting in fungal cell death. However, randomized studies produced dismal outcomes, with no statistical difference between individuals receiving laser therapy and those receiving a placebo. Lim et al. found that lasers combined with topical amorolfine improved onychomycosis after 12 weeks of treatment [43-45]. The scientists concluded that the favorable results may be due to laser-induced nail modifications, allowing for a more thorough absorption of the topical medicament. Consequently, lasers might be considered an effective therapy in older patients, patients with kidney failure or liver disorders, or patients with other abnormalities [44]. Zhong et al. [46] reported that short-pulsed 1064-nm Nd:YAG laser treatment did not improve the mycological cure rates among individuals with onychomycosis caused by *T. rubrum*. It is possible that the RCT study's extended follow-up period (12 months) resulted in a relatively high reappearance rate or that the variety of possible target chromophores decreased, affecting (reducing) the laser-tissue interaction [46]. 

However, poor prognosis of onychomycosis is associated with the burden of disease among sensitive population subgroups such as diabetic and geriatric populations. The factors behind poor prognosis are comorbidities (diabetes mellitus, immunosuppression, and peripheral vascular disease), patient characteristics (personal history of onychomycosis, older age, and exposure) [45], the severity of infecting organisms (yeasts, fungal infection, and non-dermatophytes), and nail characteristics (proximal subungual onychomycosis, severe onycholysis). Furthermore, the patients with poor prognostic factors may outweigh the risks, and the severity of onychomycosis should be monitored due to the failure of previous treatments [47]. 

Additionally, it was discovered that in many trials, individuals who had poor prognoses or risk indicators for relapse were excluded. Six laser versus topical antifungal-based studies did not give information on exclusion criteria, whereas 66.7% (6/9) of studies with exclusion requirements included a risk factor for an unfavorable outcome or high risk of relapse. As a result, published trials removed numerous individuals who might have profited the most from a combination of treatments, such as the elderly and immunodeficient, and future trials should be carried out on such groups of patients to assess the effectiveness of laser therapy in complex onychomycosis cases [48].

The efficiency of laser treatment is highly dependent on the condition of the individual and the course of therapy accomplished for recovery from onychomycosis. Carney et al. demonstrated that rubbing the diseased nail to a clear thickness of less than 2 mm before therapy proved beneficial to laser accessibility [49]. When more than 50% of the harmed nail has been impacted, inadequate nutrition of the nail and/or assault of the nail matrix can harm the long-term outcome of complying with laser therapy. With one exception of nine articles, the laser therapy was repeated at least four times. Increasing the therapy duration and the overall length of the therapy session is thought to improve the mycological cure rate and clinical effectiveness [48,49]. 

With enormous advantages, there are few limitations in the recent systematic review and meta-analysis. Firstly, there were a limited number of RCTs published on the clinical outcomes of laser therapy in comparison to other methods in the treatment of onychomycosis. Some of these were RCTs and cohort studies which may disturb the clinical outcomes of laser therapy in comparison to terbinafine or other drugs. Secondly, the recent meta-analysis and systematic review lacked the comparison among different laser therapies due to a smaller number of trials on the treatment of onychomycosis by those. Thirdly, very few trials discussed the adverse events of laser therapy in comparison to other drugs such as terbinafine. Fourthly, it is interesting to know that studies discovered that the mycological and medical effectiveness of laser treatment coupled with topical drugs was considerably greater than that of laser therapy in comparison to other drugs which attributed to the exclusion of major trials in the recent study.

## Conclusions

Laser therapy as compared to terbinafine and other topical antifungal drugs appeared to be an effective treatment option for onychomycosis with fewer adverse effects. However, the evidence is limited by the small number of comparative studies and RCTs in a recent meta-analysis. Further large-scale RCTs are recommended to evaluate the role of laser therapy as a standard treatment option for onychomycosis in comparison to other oral drugs. Additionally, long-term follow-up studies are needed to examine the treatment outcomes and potential recurrence rates among sensitive population subgroups.

## References

[1] Sigurgeirsson B, Baran R (2014). The prevalence of onychomycosis in the global population: a literature study. J Eur Acad Dermatol Venereol.

[2] Hay RJ, Baran R (2011). Onychomycosis: a proposed revision of the clinical classification. J Am Acad Dermatol.

[3] Gupta AK, Gupta G, Jain HC (2016). The prevalence of unsuspected onychomycosis and its causative organisms in a multicentre Canadian sample of 30 000 patients visiting physicians' offices. J Eur Acad Dermatol Venereol.

[4] Elewski BE, Tosti A (2015). Risk factors and comorbidities for onychomycosis: implications for treatment with topical therapy. J Clin Aesthet Dermatol.

[5] Thomas J, Jacobson GA, Narkowicz CK, Peterson GM, Burnet H, Sharpe C (2010). Toenail onychomycosis: an important global disease burden. J Clin Pharm Ther.

[6] Lipner SR, Scher RK (2019). Onychomycosis: clinical overview and diagnosis. J Am Acad Dermatol.

[7] Ghannoum MA, Salem I, Christensen L (2018). Epidemiology of onychomycosis. Onychomycosis: Diagnosis and Effective Management.

[8] Lee WJ, Kim SL, Jang YH, Lee SJ, Kim DW, Bang YJ, Jun JB (2015). Increasing prevalence of Trichophyton rubrum identified through an analysis of 115,846 cases over the last 37 years. J Korean Med Sci.

[9] Piérard GE (2006). Spores, sporodochia and fomites in onychomycosis. Dermatology.

[10] Eba M, Njunda AL, Mouliom RN, Kwenti ET, Fuh AN, Nchanji GT, Atashili J (2016). Onychomycosis in diabetic patients in Fako Division of Cameroon: prevalence, causative agents, associated factors and antifungal sensitivity patterns. BMC Res Notes.

[11] Crawford F, Young P, Godfrey C, Bell-Syer SE, Hart R, Brunt E, Russell I (2002). Oral treatments for toenail onychomycosis: a systematic review. Arch Dermatol.

[12] Gupta AK (2001). Ciclopirox nail lacquer: a brush with onychomycosis. Cutis.

[13] Grover C, Bansal S, Nanda S, Reddy BS, Kumar V (2007). Combination of surgical avulsion and topical therapy for single nail onychomycosis: a randomized controlled trial. Br J Dermatol.

[14] Lipner SR, Scher RK (2019). Onychomycosis: treatment and prevention of recurrence. J Am Acad Dermatol.

[15] Elewski BE, Rich P, Pollak R (2013). Efinaconazole 10% solution in the treatment of toenail onychomycosis: two phase III multicenter, randomized, double-blind studies. J Am Acad Dermatol.

[16] Elewski BE, Aly R, Baldwin SL (2015). Efficacy and safety of tavaborole topical solution, 5%, a novel boron-based antifungal agent, for the treatment of toenail onychomycosis: results from 2 randomized phase-III studies. J Am Acad Dermatol.

[17] Kreijkamp-Kaspers S, Hawke K, Guo L (2017). Oral antifungal medication for toenail onychomycosis. Cochrane Database Syst Rev.

[18] Darkes MJ, Scott LJ, Goa KL (2003). Terbinafine: a review of its use in onychomycosis in adults. Am J Clin Dermatol.

[19] Yan J, Wang X, Chen S (2014). Systematic review of severe acute liver injury caused by terbinafine. Int J Clin Pharm.

[20] Gupta AK, Daigle D, Foley KA (2015). Network meta-analysis of onychomycosis treatments. Skin Appendage Disord.

[21] Alster TS, Lupton JR (2001). Lasers in dermatology. An overview of types and indications. Am J Clin Dermatol.

[22] Ledon JA, Savas J, Franca K, Chacon A, Nouri K (2014). Laser and light therapy for onychomycosis: a systematic review. Lasers Med Sci.

[23] Ortiz AE, Avram MM, Wanner MA (2014). A review of lasers and light for the treatment of onychomycosis. Lasers Surg Med.

[24] Bristow IR (2014). The effectiveness of lasers in the treatment of onychomycosis: a systematic review. J Foot Ankle Res.

[25] Ma W, Si C, Kasyanju Carrero LM (2019). Laser treatment for onychomycosis: a systematic review and meta-analysis. Medicine (Baltimore).

[26] Westerberg DP, Voyack MJ (2013). Onychomycosis: current trends in diagnosis and treatment. Am Fam Physician.

[27] Page MJ, McKenzie JE, Bossuyt PM (2021). The PRISMA 2020 statement: an updated guideline for reporting systematic reviews. BMJ.

[28] Sarkis-Onofre R, Catalá-López F, Aromataris E, Lockwood C (2021). How to properly use the PRISMA statement. Syst Rev.

[29] Higgins JP, Altman DG, Gøtzsche PC (2011). The Cochrane Collaboration's tool for assessing risk of bias in randomised trials. BMJ.

[30] (2020). Welcome to RevMan 5.4. https://training.cochrane.org/system/files/uploads/protected_file/RevMan5.4_user_guide.pdf.

[31] El-Tatawy RA, Abd El-Naby NM, El-Hawary EE, Talaat RA (2015). A comparative clinical and mycological study of Nd-YAG laser versus topical terbinafine in the treatment of onychomycosis. J Dermatolog Treat.

[32] Xu Y, Miao X, Zhou B, Luo D (2014). Combined oral terbinafine and long-pulsed 1,064-nm Nd: YAG laser treatment is more effective for onychomycosis than either treatment alone. Dermatol Surg.

[33] Rajbanshi B, Shen L, Jiang M (2020). Comparative study of traditional ablative CO2 laser-assisted topical antifungal with only topical antifungal for treating onychomycosis: a multicenter study. Clin Drug Investig.

[34] Kim TI, Shin MK, Jeong KH, Suh DH, Lee SJ, Oh IH, Lee MH (2016). A randomised comparative study of 1064 nm neodymium-doped yttrium aluminium garnet (Nd:YAG) laser and topical antifungal treatment of onychomycosis. Mycoses.

[35] El-Tatawy RA, Aliweh HA, Hegab DS, Talaat RA, Shams Eldeen MA (2019). Fractional carbon dioxide laser and topical tioconazole in the treatment of fingernail onychomycosis. Lasers Med Sci.

[36] Kartik DT, Kohli S (2022). A comparative study on efficacy of fractional carbondioxide laser assisted topical antifungal therapy with topical antifungal therapy alone for treatment of onychomycosis in adult north Indian population. Int J Dermatol Venereology Leprosy Sci.

[37] Shetty P, Rangegowda SM, Vinay KN, Ravikumar BC, Nagesha PC (2023). Comparative study of safety and efficacy of combination therapy of fractional CO2 laser and topical amorolfine cream versus oral itraconazole in the treatment of onychomycosis. Lasers Med Sci.

[38] Koren A, Salameh F, Sprecher E, Artzi O (2018). Laser-assisted photodynamic therapy or laser-assisted amorolfine lacquer delivery for treatment of toenail onychomycosis: an open-label comparative study. Acta Derm Venereol.

[39] Lu S, Zhang J, Liang Y, Li X, Cai W, Xi L (2016). The efficacy and prognostic factors for long pulse neodymium: yttrium-aluminum-garnet laser treatment on onychomycosis: a pilot study. Ann Dermatol.

[40] Yeung K, Ortner VK, Martinussen T, Paasch U, Haedersdal M (2019). Efficacy of laser treatment for onychomycotic nails: a systematic review and meta-analysis of prospective clinical trials. Lasers Med Sci.

[41] Gupta AK, Surprenant MS, Kempers SE, Pariser DM, Rensfeldt K, Tavakkol A (2021). Efficacy and safety of topical terbinafine 10% solution (MOB-015) in the treatment of mild to moderate distal subungual onychomycosis: a randomized, multicenter, double-blind, vehicle-controlled phase 3 study. J Am Acad Dermatol.

[42] Gupta AK, Foley KA, Mays RR, Shear NH, Piguet V (2020). Monotherapy for toenail onychomycosis: a systematic review and network meta-analysis. Br J Dermatol.

[43] Lim EH, Kim HR, Park YO (2014). Toenail onychomycosis treated with a fractional carbon-dioxide laser and topical antifungal cream. J Am Acad Dermatol.

[44] Ferwerda B, Ferwerda G, Plantinga TS (2009). Human dectin-1 deficiency and mucocutaneous fungal infections. N Engl J Med.

[45] Hochman LG (2011). Laser treatment of onychomycosis using a novel 0.65-millisecond pulsed Nd:YAG 1064-nm laser. J Cosmet Laser Ther.

[46] Zhong S, Lin GT, Zhao JY (2019). Efficacy of two-stage treatment of onychomycosis using a long-pulsed Nd:YAG 1064-nm laser. Evid Based Complement Alternat Med.

[47] Yang Y, Liu H, Yang RY (2015). The observation of efficacy of Ultrapulse CO2 fractional laser treatment for onychomycosis. Chin J Dermatol.

[48] Zhou BR, Lu Y, Permatasari F (2016). The efficacy of fractional carbon dioxide (CO2) laser combined with luliconazole 1% cream for the treatment of onychomycosis: a randomized, controlled trial. Medicine (Baltimore).

[49] Carney C, Cantrell W, Warner J, Elewski B (2013). Treatment of onychomycosis using a submillisecond 1064-nm neodymium:yttrium-aluminum-garnet laser. J Am Acad Dermatol.

